# Beam narrowing test: a motor index of post-stroke motor evaluation in an aged rat model of cerebral ischemia

**DOI:** 10.1007/s00702-024-02768-0

**Published:** 2024-04-10

**Authors:** Leonard Radu Pinosanu, Ianis Kevyn Stefan Boboc, Tudor Adrian Balseanu, Andrei Gresita, Dirk M. Hermann, Aurel Popa‐Wagner, Bogdan Catalin

**Affiliations:** 1 Chair of Vascular Neurology, Dementia and Ageing, University Hospital Essen, University of Duisburg-Essen, 45147 Essen, Germany; 2 Department of Pharmacology, University of Medicine and Pharmacy of Craiova, 200349 Craiova, Romania; 3 Experimental Research Centre for Normal and Pathological Aging, University of Medicine and Pharmacy of Craiova, 200349 Craiova, Romania; 4 Department of Biomedical Sciences, New York Institute of Technology, College of Osteopathic Medicine, Old Westbury, NY 115680-8000 USA

**Keywords:** Aging, Stroke, Rats, Behaviour, Beam narrowing test, Motor performance index

## Abstract

Each year, 15 million people worldwide suffer from strokes. Consequently, researchers face increasing pressure to develop reliable behavioural tests for assessing functional recovery after a stroke. Our aim was to establish a new motor performance index that can be used to evaluate post-stroke recovery in both young and aged animals. Furthermore, we validate the proposed procedure and recommend the necessary number of animals for experimental stroke studies. Young (n = 20) and aged (n = 27) Sprague–Dawley rats were randomly assigned to receive either sham or stroke surgery. The newly proposed performance index was calculated for the post-stroke acute, subacute and chronic phases. The advantage of using our test over current tests lies in the fact that the newly proposed motor index test evaluates not only the performance of the unaffected side in comparison to the affected one but also assesses overall performance by taking into account speed and coordination. Moreover, it reduces the number of animals needed to achieve a statistical power of 80%. This aspect is particularly crucial when studying aged rodents. Our approach can be used to monitor and assess the effectiveness of stroke therapies in experimental models using aged animals.

**Supplementary Information:**

The online version contains supplementary material available at 10.1007/s00702-024-02768-0.

## Introduction

Worldwide, 15 million people suffer a stroke each year. Of these, 5 million die, and another 5 million remain permanently disabled. Consequently, stroke is associated with substantial economic and social costs WHO EMRO 2023). In 2017, the global cost of stroke amounted to approximately 451 billion dollars, with around 89% of stroke patients living in underdeveloped or developing countries. For most stroke patients, treatment is still limited to supportive care only. As a result, researchers are under increasing pressure to identify new molecular pathways that could be used as effective therapies (Lakhan et al. 2009) or aid in enhancing recovery outcomes.

Despite significant research investment, the only available therapeutic options are mechanical thrombectomy and tissue plasminogen activator thrombolysis. None of the over one thousand drugs tested on animal models have been successful in human clinical trials. Therefore, there is an urgent need to identify effective therapies for both preventing cerebral ischemia and enhancing stroke recovery.

Because stroke primarily affects individuals with co-morbidities, it is especially relevant to evaluate the efficacy of stroke medications using an appropriate animal stroke model. Unfortunately, these characteristics are commonly overlooked in animal models of stroke (Roy-O’Reilly and McCullough 2018), which may explain why all medications shown to be successful in juvenile animal models have failed in human clinical trials (Boboc et al. 2023; Carmichael 2005; Lyden 2021).

In rat models of stroke, neurobehavioural evaluations aim to measure the post-stroke recovery of sensorimotor deficits, cognition, and memory (Boyko et al. 2011; Ruan and Yao 2020). The rotating pole and the foot fault test are employed to evaluate motor coordination and balance, while the corner test assesses sensorimotor deficits. The adhesive removal test evaluates somatosensory and motor deficits, and The cylinder test assesses post-stroke forelimb placement asymmetry. Spatial learning and memory deficits are assessed using the T-Maze test, which evaluates spatial working memory. Finally, some researchers prefer a Modified Neurological Severity Score (mNSS), combining various assessments to provide a composite score that evaluates neurological function aspects.

Aging is the most important risk factor for stroke. For this reason, the STAIR committee recommends that all drugs be tested on aged animals, and that behavioral testing should also be conducted on aged animals (Modo et al. 2018). Indeed, as we and a few others have shown, functional recovery after stroke in aged rodents follows a quite different pattern in terms of both molecular and cellular events and behavioural recovery (Hermann et al. 2019a, b; Popa-Wagner et al. 2014).

In previous research, the sensorimotor functions of both forelimbs and hindlimbs were tested in young and aged post-stroke rats. The rats were treated with galantamine, a selective competitive cholinesterase inhibitor, using a tapered/ridged beam (Zhao et al. 2005). In this study, we provide a detailed description of the procedure, as well as acute, subacute and chronic results for both young and aged animals, enabling us to objectively measure the motor abilities of post-stroke animals. Additionally, we validated the proposed standardized behaviour test and determined the minimum number of animals required to achieve statistical significance.

## Materials and methods

### Animals

All experiments described were performed on animals, with both young (3–5 months) and aged (20–22 months) Sprague–Dawley rats. Based on our experience, 17 (weight: 500–650 g) aged animals will be required per group for the behavioural analysis of neurological recovery after stroke. This calculation is founded on an expected effect size of 25% of mean values, which will yield statistically significant effects (α error < 0.05) with a power of 0.80. This assumption considers that common standard deviations are 25% of mean values. Given the smaller parameter variability in young and sham animals, we opted for 10 animals per group for both young sham (weight: 310–400 g), aged sham (weight: 500–650 g) and young animals (weight: 310–400 g).

The animals were kept on a 12 h light/dark cycle in a temperature-controlled room with free access to water and food. After randomization, the animals were transferred to the testing location and given 48 h to acclimate. All experiments were approved by the Institutional Animal Care and Use Committee of the University of Medicine and Pharmacy Craiova (approval nr. 2.5 from 29.10.2020), and by the National Sanitary Veterinary and Food Safety Authority Dolj (approval nr. 12 from 29.03.2022). All animal experiments complied with the ARRIVE guidelines and were carried out in accordance with the EU Directive 2010/63/EU for animal experiments.

### Beam narrowing walking test

All behavioural tests were performed by two investigators blinded to the identity of the animal groups.

### Animal training

First, the animals were given three to five days of training. This involved positioning the animals on a narrowing beam 135 cm in length and encouraging them to walk towards the end of the beam for three to five trials until continuous walking was achieved. During the training period the goal is to have animals capable of walking, without stopping, from one part to another of the beam. While some animals have a good performance from the start, other animals are more anxious and will need more patience and encouragement. To minimize the stress of the animals we recommend the first placement of the animal, within the testing period, to be in front of the resting chamber and not at the starting position, in order for the animal to easily be able to enter the “safe space”. The placement distance should gradually be increased, either from trial to trial or day by day, depending on the performance of each animal. Regardless of the individual variability, young animals perform the task within 3 days, while older animals will be able to perform it with around 5 days of training. Once the animal reached the resting cage, a period of at least 30 s was allowed between trials. This period of relaxation should not exceed one minute before the start of a new trial or before the animal was returned to its normal environment. To further encourage the animals to reach the resting area, small amounts of peanut butter were concealed within the cage. Following the examination of each animal, the apparatus was disinfected with 70% ethanol. Animals unable to perform this task within 3 days for young animals and within 5 days for aged animals should be excluded from the study group.

### Recording the test

**Fig. 1:**
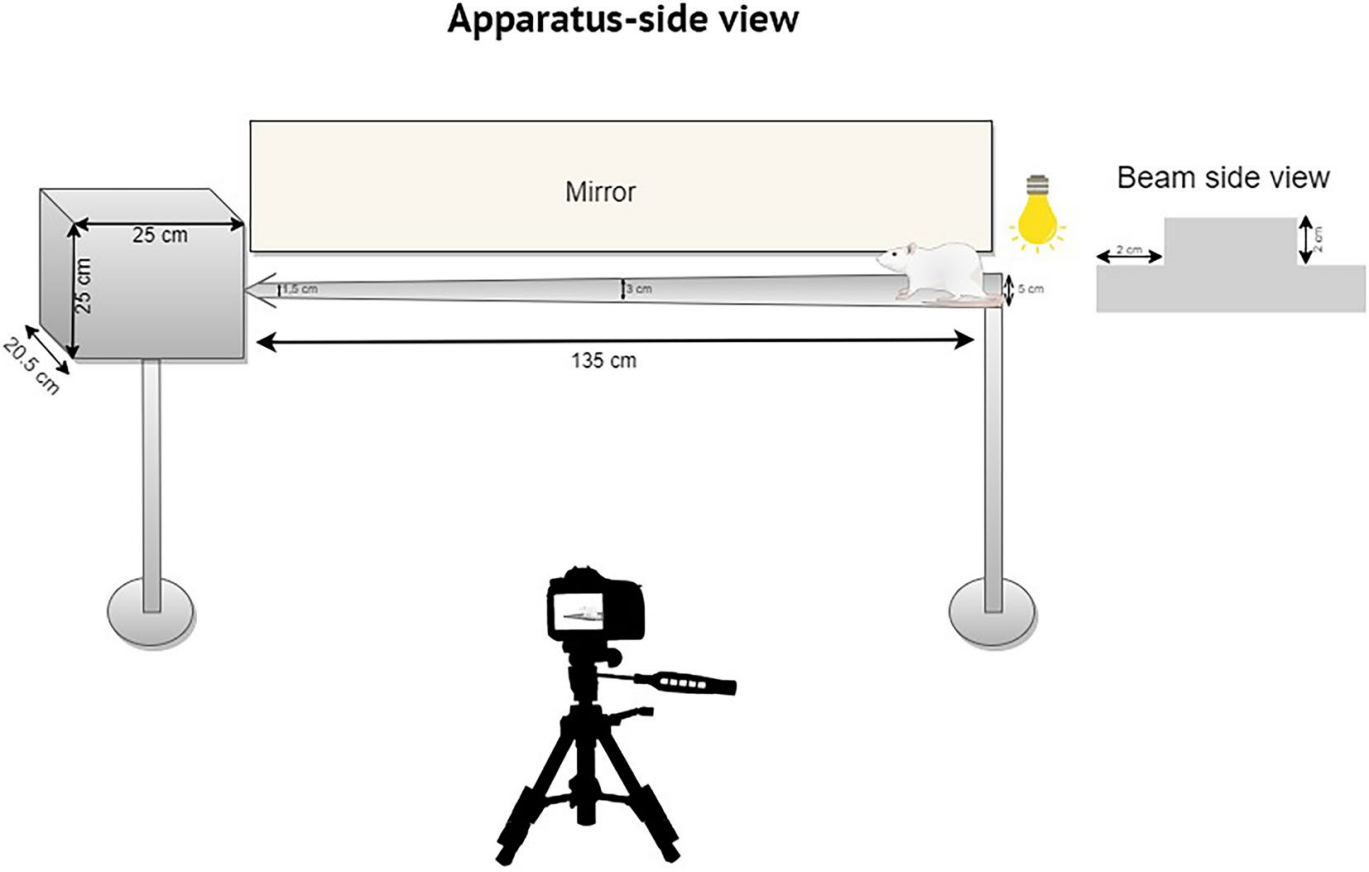
Narrow beam apparatus: side view of the beam apparatus and side view of the wooden beam. A fixed camera was used to record the narrowing beam crossing, which is 1.35 m in length, with a starting width of 5 cm and a finishing width of 1.5 cm

When an animal was able to cross the beam without hesitation, it was considered as being trained. Due to the shape of the beam (Fig. 1), at the starting point the animals had a large area (5 cm width) to step on. However, once the animal begins to move toward the resting area, the beam’s width progressively decreases to 3 cm in the beam’s center and 1.5 cm just before the resting chamber. The beam and the resting cage are placed 50 cm above the ground. A mirror was also conveniently placed to enable simultaneous observation of all four paws. A video camera was used to record the trial. At the beginning of each trial, a bright light positioned above the starting point was activated to encourage the rats to cross the beam (Fig. 1).

### Evaluation of the test

**Fig. 2:**
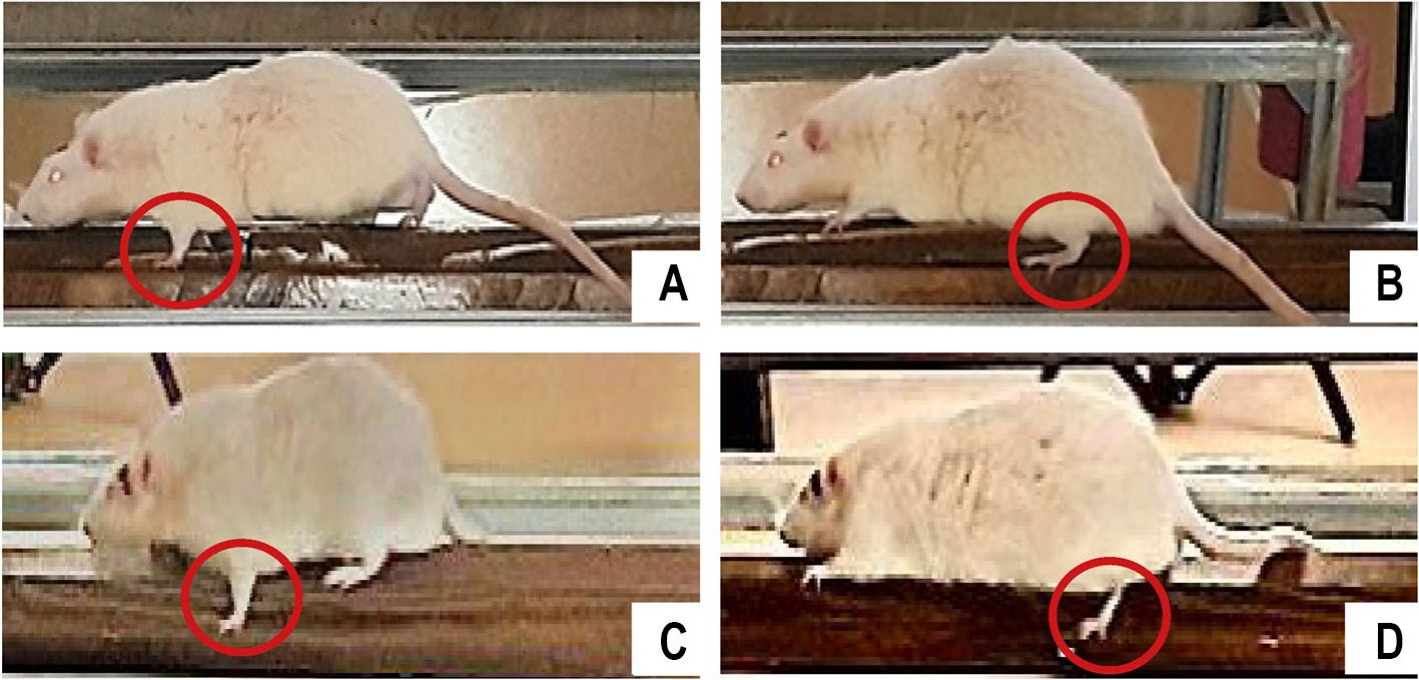
Examples of experimental animals misstepping during the recording. Due to the setup, the beam causes the left front paw to slip (**A**), the left back paw to slip (**B**), the right front paw to slip (**C**), and the right back paw to slip (**D**)

Animal performance was recorded and analyzed. For each trial, the number of missteps for each paw (Fig. 2) and the crossing time were recorded. The average value of each parameter over the course of each day was used in the analysis. At the end of the day, a performance index was calculated using the average time required to complete the test, multiplied by the arithmetic mean of the slips made by the animal for each member (Table 1). A lower index reflects better performance. For each individual, the data obtained were used to calculate the daily performance index using the following formula:

$$\left( {\sum \overline{Mistakes/paw} *1/4} \right)*\left( {\overline{Crossing \,time} } \right)$$

**Table 1:** Example of results obtained for one animal

Animal No. 1	Trial 1	Trial 2	Trial 3	Arithmetic mean	Performance index
Crossing time (CT)	4	4	6	4.66	3.88
Right front paw slips (RF)	0	0	3	1	
Left front paw slips (LF)	3	0	0	1	
Right back paw slips (RB)	0	1	2	1	
Left back paw slips (LB)	0	0	1	0.33	

**Fig. 3:**
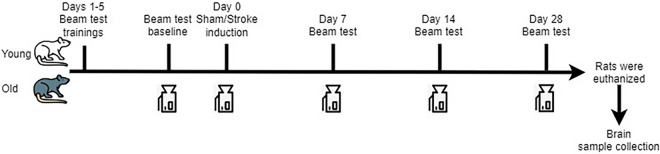
Timeline of the Motor Performance Index experiment. After acclimatization and training, a baseline was established for all animals used in the current experiment. Under identical recording conditions, testing was repeated at 7, 14, and 28 days post-stroke

Except for the training sessions, all other trials were recorded. Prior to stroke surgery, the baseline performance index was calculated for each animal. Following the stroke surgery, behavioural evaluations were conducted every 7 days until day 28, at which point the animals were euthanized, and their brains were analyzed (Fig. 3).

### Induction of focal cerebral ischemia

All animals were subjected to either middle cerebral artery occlusion (MCAo) or sham surgery. MCAo was induced as previously described (Gresita et al. 2022). In brief, after anesthesia (I.P. mix of ketamine (60 mg/kg) and xylazine (8 mg/kg)), a small craniotomy was made above the right somatosensory cortex. Using a laser Doppler device (Perimed, Stockholm, Sweden), the normal blood flow was measured for 20 s. Following this, the right MCA was thermocoagulated. The occlusion was first visually confirmed, and then the blood flow was measured and compared to the normal flow. An 80% drop in blood flow was considered successful. Bone wax was used to seal the brain, and the muscle/skin was sutured. Throughout the procedure, the body temperature of the animal was maintained at 37 °C using a Homeothermic Blanket System (Harvard Apparatus).

### Infarct volume determination

At the end of the experiment, the animals were anesthetized using a mix of Ketamine and Xylazine (60 mg/kg, 8 mg/kg), and perfused with neutral buffered saline followed by buffered 4% freshly depolymerized paraformaldehyde. The brain was removed, post-fixed in 4% buffered paraformaldehyde for 24 h, cryoprotected in 15% glycerol prepared in 10 mmol/l phosphate buffered saline, flash-frozen in isopentane, and stored at − 70 °C until sectioning.

To assess the size of the infarct induced by permanent focal cerebral ischemia, brain sections at 500 µm intervals (i.e., every 20th section) were stained with methyl green/pyronine Y. Images of the stained sections were captured, and the infarct areas were measured using ImageJ. Infarct areas at various rostrocaudal levels were manually delineated and used to calculate partial infarct volumes by multiplying by the section thickness and accounting for the number of discarded sections in between. By integrating partial infarct volumes across the brain, the total infarct volume was determined.

### Power and sample size determination

The power of the performance index and the sample size were calculated using GraphPad StatMate 2.00 for Windows (GraphPad Software, www.graphpad.com). We were able to determine the power of the experiment through this software. For this purpose, we employed an unpaired t-test approach that utilized the numbers of animals and their standard deviation for each group. By identifying the difference between the means of the Sham and MCAo groups, we determined the power for a significance level of 0.05 in a two-tailed analysis. The normal Gaussian distribution of animal scores was verified using the Kolmogorov–Smirnov test. We plotted the data for a power percentage interval ranging from 10 to 99%. Using the same software, we determined the future sample size, assuming the use of an unpaired t-test by comparing two means based on the differences between the standard deviations of each group, with a significance level of 0.05, two-tailed.

## Results

### Infarct volume

**Fig. 4:**
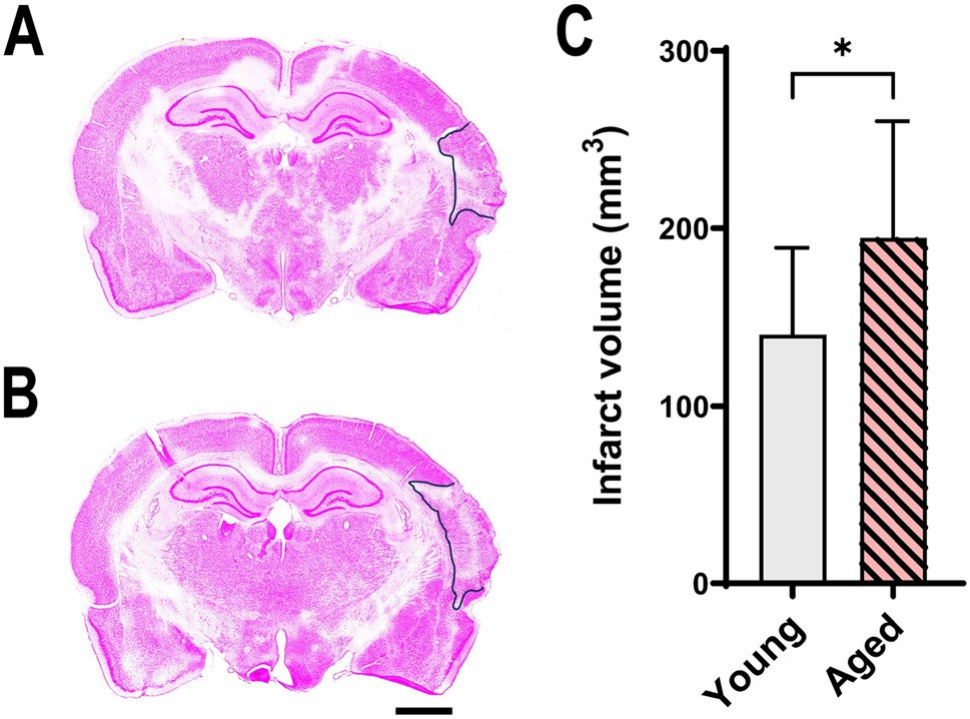
Representative images of the infarct at 28 days post-stroke in young (**A**) and aged (**B**) animals. Young animals, N = 10; aged animals, N = 17. Data is shown as Mean ± SD (**C**). Bar scale: 1000 μm

Stroke volume at 28 days was significantly higher in aged animals (167.81 ± 75.85) compared with young animals (146.54 ± 58.46), p = 0.047 (Fig. 4).

### Motor performance index by beam narrowing test is able to detect differences in acute and chronic phases of MCAo

**Fig. 5:**
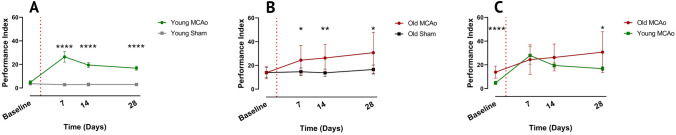
Motor Performance Index in Young Sham vs. Young MCAo Animals (**A**). **B** Performance Index in Aged Sham vs. Aged MCAo Animals. **C** Performance Index in Aged MCAo Animals vs. Young MCAo Animals. Data are presented as mean ± SD. Young animals: Sham, N = 10; MCAo, N = 10; Aged animals: Sham, N = 10, MCAo, N = 17

The motor performance index in the beam narrowing test did not detect movement impairments in young and aged Sham animals (Supplementary Fig. 1). Of note, acute post-stroke juvenile animals performed worse on the beam narrowing test (26.40 ± 4.50) than Shams (2.79 ± 1.20) (p < 0.0001). At seven days post-stroke, the recorded mean difference between the two groups was 23.60. We noted the low standard deviation shown by data in the young group. The tests performed during the subacute post-stroke period indicated a performance index of 19.85 ± 4.51 compared to 2.96 ± 1.36 in Sham animals (p < 0.0001) (Fig. 5A). The mean performance index remained high at 28 days post-stroke (16.38 ± 5.186) as compared to Shams (2.95 ± 0.85) (p < 0.0001). We then used the performance index to establish differences between Sham and MCAo aged animals during the acute (14.62 ± 3.25 compared to 24.44 ± 12.53) (p = 0.025), subacute (13.73 ± 3.13 compared to 26.23 ± 11.33) (p = 0.001), and chronic phase of stroke (16.51 ± 3.8 compared to 30.71 ± 17.32) (p = 0.017) (Fig. 5B).

The performance index measured with the beam narrowing test is age-dependent. According to the average performance index, at baseline, the older sham rats perform worse than their younger counterparts (13.92 ± 4.25 compared to 3.66 ± 0.68) (p < 0.0001) (Supplementary Fig. 1). MCAo surgery worsens the performance index for both young (26.4 ± 4.5) and aged (24.44 ± 12.52) animals during the acute phase (p > 0.05) (Fig. 5C). No significant difference is observed between young (19.85 ± 4.1) and aged (26.23 ± 11.33) animals during the subacute phase. However, the performance index reveals that young animals recover significantly better and more quickly (16.36 ± 5.1) than older animals during the chronic phase (30.71 ± 17.32) (p = 0.018) (Fig. 5C).

### The power and sample size needed for measuring the performance index

**Fig. 6:**
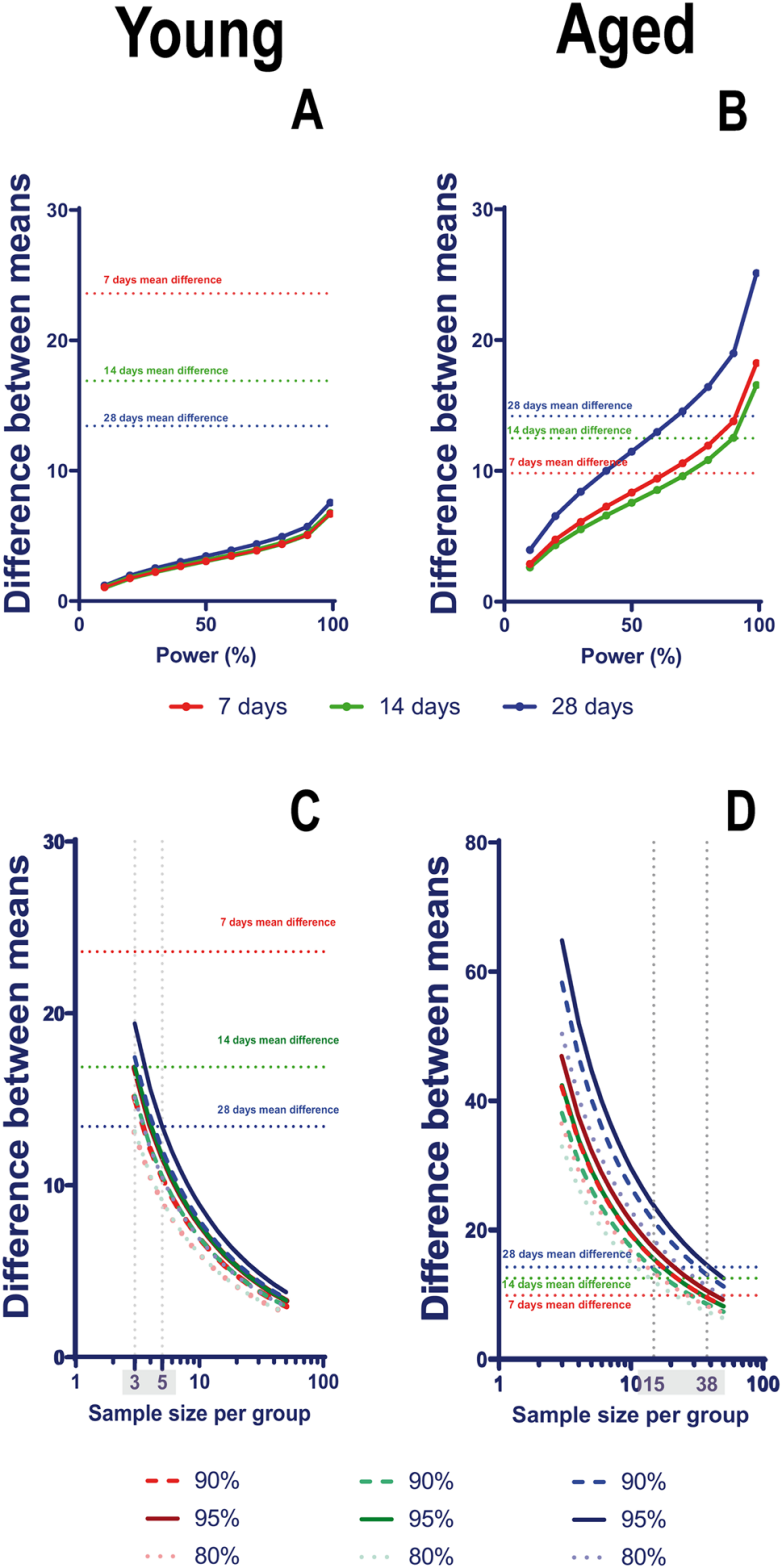
Power and sample size determination were performed for both young and aged animals. The mean difference between Sham and MCAo animals was calculated at each timepoint. By plotting the powers for 7 (red line), 14 (green line), and 28 (blue line) days, we demonstrated that the differences obtained with the proposed performance index (indicated by red, green, and blue dotted lines) achieved a power of 99% for young MCAo animals compared to Sham animals (**A**). For aged MCAo animals, the power of the Performance Index test was initially low (63.6%), but it increased to 89.8% during the subacute phase of stroke (**B**). The minimum number of young MCAo animals required for future experiments was determined for power levels of 80%, 90%, and 95%, at each timepoint, indicating that 3–5 young animals are sufficient for the proposed performance index to achieve significance (**C**). Conversely, the minimum number of aged MCAo animals required for future experiments to achieve power levels of 80%, 90%, or 95% is significantly higher compared to young animals, requiring between 15 and 38 animals, depending on the desired power and the timepoint under investigation (**D**)

We assessed the ability of the performance index to differentiate between young and aged Sham and MCAo animals during acute, subacute, and chronic post-stroke periods. Taking into account the differences in means at 7 (23.60), 14 (16.89), and 28 days (13.43) post-stroke, the p values are “statistically significant” in 95.9% of replicated experiments (using a two-tailed experimental setup) (Fig. 6A). In the acute phase, the effectiveness of this index decreased for older animals, scoring only 63.6% (Fig. 6B). Interestingly, the power increased to 89.8% during the subacute phase and then decreased to 67.8% during the chronic phase (Fig. 6B). In summary, the group size required for young animals to achieve statistical significance at day 28 is 8, while the group size required for aged animals to achieve statistical significance at day 28 is 24 (Fig. 6C, D) (G*Power analysis, Supplementary Tables).

## Discussion

The annual number and severity of strokes have substantially increased from 1990 to 2019, and it is estimated that the economic and social burdens induced by stroke will continue to grow across the world, particularly in low-income countries (Feigin et al. 2022). Due to the increased social and economic burdens, there is a growing demand for research into strokes. While clinical research can address the implications of stroke-causing and aggravating factors, the vast majority of preclinical research focuses on cellular, molecular, and genetic approaches in an effort to thoroughly elucidate the pathophysiological mechanisms and identify the most effective therapies for stroke (Herson and Traystman 2014).

Despite the fact that some studies have described the cells (Ma et al. 2021; Sekerdag et al. 2018) and molecular pathways (Turley et al. 2009) implicated in stroke, there is still a need for precise systemic output measurements of behavioural outcomes. Consequently, animal testing is essential to assess the efficacy of any drug- or cell-based therapy (Balseanu et al. 2014; Bulygin et al. 2020; Surugiu et al. 2019).

The majority of behavioural tests, however, are not standardized due to modifications made to the original method, making it virtually impossible to compare data across articles (Boboc et al. 2023). A recent meta-analysis emphasized the lack of standardization (Boboc et al. 2023), likely as a result of the difficulty in implementing tests that require expensive equipment (Garrick et al. 2021) or a substantial amount of time from the researcher in preparing the tested animals (Boboc et al. 2023).

The animals’ proficiency in the beam narrowing test exploits the coordination and balance deficiencies of MCAo animals (Luong et al. 2011). The beam narrowing test can detect minute differences (Luong et al. 2011), but individual characteristics such as the strength of the animals, their mood, and their level of fatigue can all affect the ultimate result. Here, we propose the implementation of an easily calculable performance index for the beam narrowing test (Zhao et al. 2005), which can mitigate a portion of the individual variance. The materials required to construct the scaffold and the proposed algorithm provide a precise and viable alternative to other motor tests.

Throughout the experiment, it was observed that the sham surgery animals had a consistent performance index, with older animals demonstrating a statistically significant motor deficit compared to younger animals (Supplementary Fig. 1). During the acute post-stroke period, the performance index of young rats exposed to MCAo declines from (4.82 ± 1.41) to (26.40 ± 4.50) (p < 0.0001) (Fig. 5A). During the subacute phase, the performance index of young rats improved to 19.85 ± 4.51 (p < 0.0001) at 14 days, and to 16.38 ± 5.18 (p < 0.0001) during the chronic phase (Fig. 5A).

The proposed algorithm reflects the slow recovery of aged animals, with the acute performance index of this group reaching a value of (24.44 ± 12.52) (p < 0.05) (Fig. 5B), comparable to that of young animals in the same phase (26.40 ± 4.50) (p > 0.05) (Fig. 5C). Although the performance index did not find any differences between young and aged animals in the subacute period (19.85 ± 4.51 vs. 26.24 ± 11.33) (p > 0.05) (Fig. 5C), the aged animals were unable to recover spontaneously, allowing the performance index to detect differences between the two groups in the chronic phase (16.38 ± 5.18 vs. 30.71 ± 17.32) (p < 0.05) (Fig. 5C).

Because age can influence stroke prognosis (Ma et al. 2020), aged animals exhibited a higher stroke volume (167.81 ± 75.85) compared to young animals (146.54 ± 58.46), p = 0.047 (Fig. 4), which was reflected in the inferior performance of older animals during recovery.

There is increasing pressure to utilize the minimum number of animals possible due to animal welfare concerns. Therefore, power and sample size determination were performed for both young and aged animals and the mean difference between Sham and MCAo animals was calculated at each timepoint. By plotting the powers for 7 (red line), 14 (green line), and 28 (blue line) days, we demonstrated that the differences obtained with the proposed performance index (indicated by red, green, and blue dotted lines) achieved a power of 99% for young MCAo animals compared to Sham animals (Fig. 6A). For aged MCAo animals, the power of the Motor Index test was initially low (63.6%), but it increased to 89.8% during the subacute phase of stroke (Fig. 6B). The minimum number of young MCAo animals required for future experiments was determined for power levels of 80%, 90%, and 95%, at each timepoint, indicating that 3–5 young animals are sufficient for the proposed performance index to achieve significance (Fig. 6C). Conversely, the minimum number of aged MCAo animals required for future experiments to achieve power levels of 80%, 90%, or 95% is significantly higher compared to young animals, requiring between 15 and 38 animals, depending on the desired power and the timepoint under investigation (Fig. 6D).

## Conclusions

In a rat model of MCAo, the performance index calculated for the beam-narrowing test represents an objective method for assessing post-stroke behaviour. The proposed parameter enables standardization and interlaboratory result comparison at a low manufacturing cost. The index can assess motor impairment in all phases of stroke in an age-dependent manner, making it a useful tool for distinguishing between Sham and MCAo animals. The advantage of using our test over the original one proposed by Tim Schallert and his colleagues (Schallert et al. 2000) is that it allows for a more comprehensive evaluation. In addition to comparing the lesioned side with the unlesioned one, our motor test evaluates not only the performance of the unaffected side compared to the affected one across three different segments of the route but also assesses overall performance by considering speed and coordination. This is especially important when studying aged rodents. Secondly, our test can be employed to monitor motor recovery in very old post-stroke rats. Thirdly, the proposed index exhibits a significantly greater increase across the acute, subacute, and chronic stroke phases compared to the standard test currently in use (see Supplementary Fig. 2A). Additionally, the proposed index demonstrates less variability than the original version when applied to aged animals (see Supplementary Fig. 2B). Finally, our motor index will facilitate a reduction in the number of sham and stroke procedures required for young animals in future experiments. Consequently, the minimum number of young animals needed post-stroke for future experiments ranges from three to five, depending on the post-stroke time point analyzed, in order to achieve 80% statistical power. For aged animals, the minimum number required to achieve 80% power 14 days after the stroke is significantly higher, at fifteen. It should also be noted that post-stroke behavioral tests are affected by stroke surgery, post-operative care, and behavioral compensation due to repeated testing. Therefore, there is a debate regarding which test to use in long-term stroke recovery studies to minimize false-positive results by selecting an appropriate task or a battery of tasks (Balkaya et al. 2018).

### Supplementary Information

Below is the link to the electronic supplementary material.

Supplementary file1 (DOCX 332 kb)

## Data Availability

The data that support the findings of this study are available from the corresponding author, upon reasonable request.
