# Supplementary material for: Beam narrowing test: a motor index of post-stroke motor evaluation in an aged rat model of cerebral ischemia
Source: J Neural Transm (Vienna). 2024 Apr 10;131(7):763–71. doi: 10.1007/s00702-024-02768-0 (PMC11199207; doi:10.1007/s00702-024-02768-0)
Supplement: Supplementary file 1 — Supplementary file1 (DOCX 332 kb) [file 702_2024_2768_MOESM1_ESM.docx]

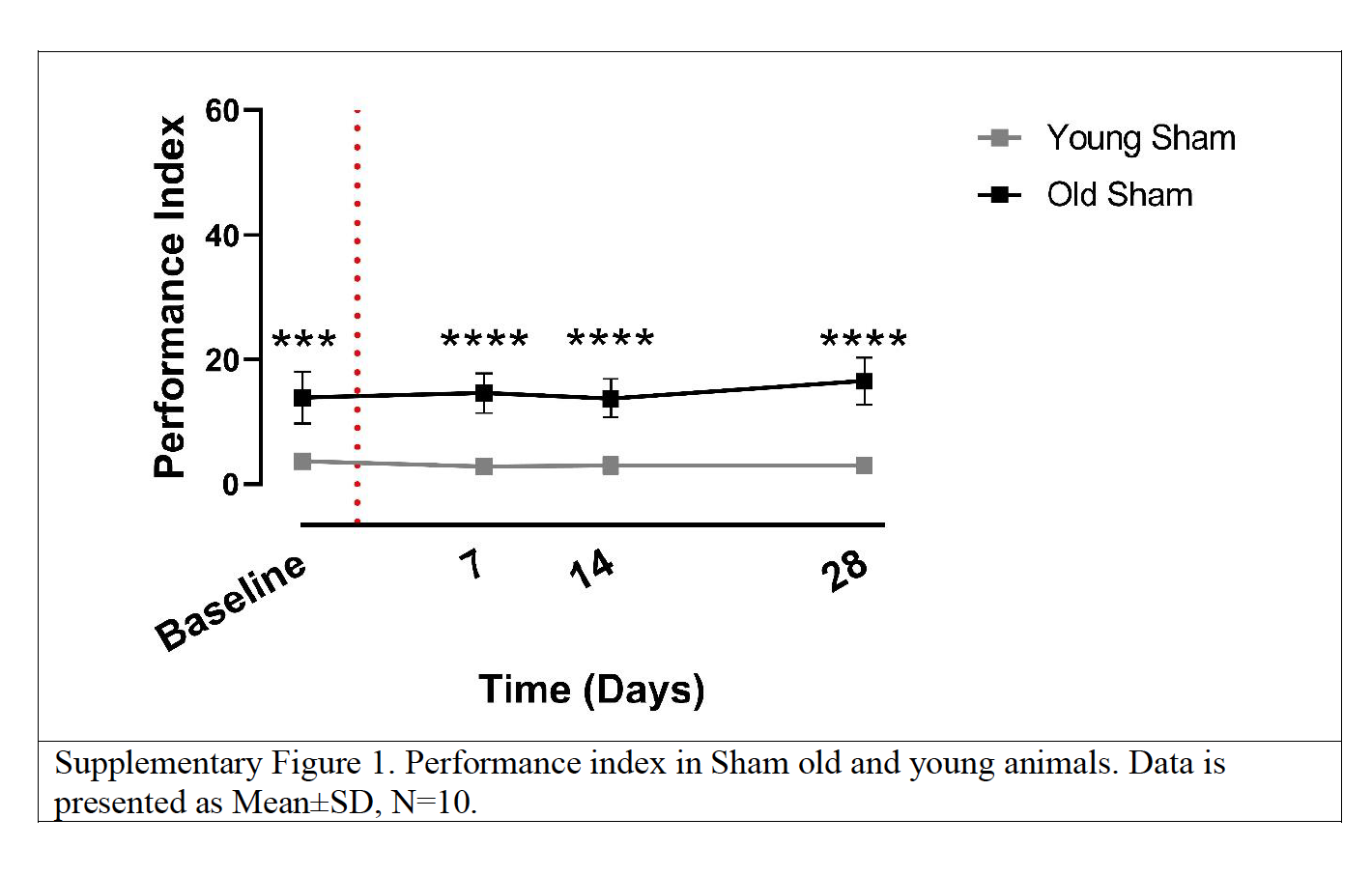


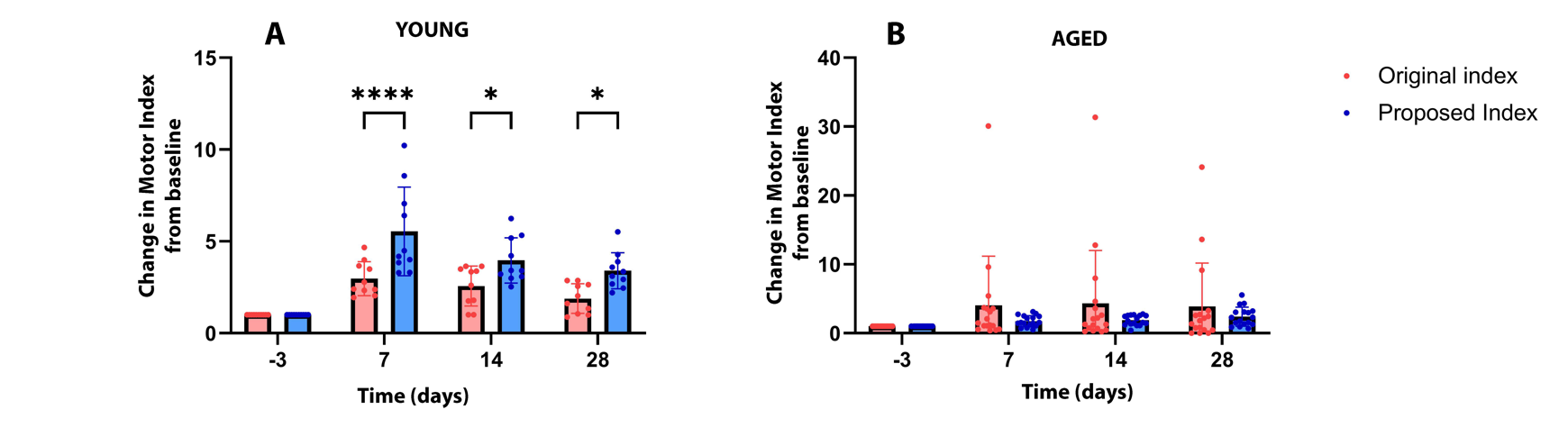


**Supplementary Figure 2. Comparison of the original and proposed indices in young and aged animals**. Both indices detected performance worsening in young MCAo animals. However, the proposed index shows a significantly greater increase across acute, subacute, and chronic stroke phases (**A**). The proposed index also demonstrates less variability than the original version when applied to aged animals (**B**).

**Tables 1-5s. Motor index and sample size determination for young and aged animals to reach the desired significance of power at different time points after stroke**

| Days | Young Sham SD | Young MCAo SD | Dif Mean | Power % |
| --- | --- | --- | --- | --- |
| Baseline | 0.68 | 1.40 | 1.19 | 75 |
| 7 | 1.20 | 4.50 | 23.60 | 99.99 |
| 14 | 1.36 | 4.51 | 16.89 | 99.99 |
| 28 | 0.85 | 5.18 | 13.43 | 99.99 |
|  | | | | |
|  | Aged Sham SD | Aged MCAo SD | Dif Mean | Power % |
| Baseline | 4.25 | 5.07 | -0.08 | 2.2 |
| 7 | 3.25 | 12.52 | 9.82 | 63.6 |
| 14 | 3.13 | 11.33 | 12.49 | 89.8 |
| 28 | 3.81 | 17.32 | 14.20 | 67.8 |
|  | | | | |
|  | Young MCAo SD | Aged MCAo SD | Dif Mean | Power % |
| Baseline | 1.41 | 5.07 | 9.01 | 99.99 |
| 7 | 4.50 | 12.52 | -1.95 | 0.80 |
| 14 | 4.51 | 11.33 | 6.38 | 36.5 |
| 28 | 5.18 | 17.32 | 14.33 | 67.6 |

| Days | Young Sham SD | Young MCAo SD | | **N** | | **Power %** | | | | | | | | | | | | | | | | | | | | | | | |  |
| --- | --- | --- | --- | --- | --- | --- | --- | --- | --- | --- | --- | --- | --- | --- | --- | --- | --- | --- | --- | --- | --- | --- | --- | --- | --- | --- | --- | --- | --- | --- |
|  |  |  |  |  |  | **7 days** | | | | | | | | **14 days** | | | | | | | | **28 days** | | | | | | | |  |
|  |  |  |  |  |  | **23.60** | | | | | | | | **16.89** | | | | | | | | **13.43** | | | | | | | |  |
| 7 | 1.20 | 4.50 | |  |  | **95%** | | **90%** | | **85%** | | **80%** | | **95%** | | **90%** | | **85%** | | **80%** | | **95%** | | **90%** | | **85%** | | **80%** | |  |
| 14 | 1.36 | 4.51 | | **3** | | 16.85 | | 15.15 | | 14.01 | | 13.10 | | 16.9 | | 15.2 | | 14.04 | | 13.13 | | 19.42 | | 17.46 | | 16.12 | | 15.08 | |  |
| 28 | 0.85 | 5.18 | | 4 | | 13.55 | | 12.19 | | 11.26 | | 10.53 | | 13.59 | | 12.22 | | 11.29 | | 10.56 | | 15.62 | | 14.04 | | 12.97 | | 12.12 | |  |
|  |  |  | | 5 | | 11.66 | | 10.48 | | 9.69 | | 9.06 | | 11.69 | | 10.51 | | 9.71 | | 9.08 | | 13.43 | | 12.08 | | 11.15 | | 10.43 | |  |
|  |  |  | | 6 | | 10.39 | | 9.34 | | 8.63 | | 8.07 | | 10.42 | | 9.37 | | 8.65 | | 8.09 | | 11.97 | | 10.76 | | 9.94 | | 9.29 | |  |
|  |  |  | | 7 | | 9.46 | | 8.5 | | 7.86 | | 7.35 | | 9.49 | | 8.53 | | 7.88 | | 7.37 | | 10.9 | | 9.8 | | 9.05 | | 8.46 | |  |
|  |  |  | | 8 | | 8.74 | | 7.86 | | 7.27 | | 6.79 | | 8.77 | | 7.89 | | 7.28 | | 6.81 | | 10.07 | | 9.06 | | 8.36 | | 7.82 | |  |
|  |  |  | | 9 | | 8.17 | | 7.34 | | 6.79 | | 6.35 | | 8.19 | | 7.37 | | 6.80 | | 6.36 | | 9.41 | | 8.46 | | 7.81 | | 7.31 | |  |
|  |  |  | | 10 | | 7.69 | | 6.92 | | 6.39 | | 5.98 | | 7.72 | | 6.94 | | 6.41 | | 5.99 | | 8.86 | | 7.97 | | 7.36 | | 6.88 | |  |
|  |  |  | | 12 | | 6.95 | | 6.25 | | 5.78 | | 5.40 | | 6.97 | | 6.27 | | 5.79 | | 5.41 | | 8.01 | | 7.2 | | 6.65 | | 6.22 | |  |
|  |  |  | | 14 | | 6.39 | | 5.74 | | 5.31 | | 4.96 | | 6.41 | | 5.76 | | 5.32 | | 4.98 | | 7.36 | | 6.62 | | 6.11 | | 5.71 | |  |
|  |  |  | | 16 | | 5.94 | | 5.34 | | 4.94 | | 4.62 | | 5.96 | | 5.36 | | 4.95 | | 4.63 | | 6.85 | | 6.16 | | 5.69 | | 5.32 | |  |
|  |  |  | | 18 | | 5.58 | | 5.02 | | 4.64 | | 4.34 | | 5.6 | | 5.03 | | 4.65 | | 4.35 | | 6.43 | | 5.78 | | 5.34 | | 4.99 | |  |
|  |  |  | | 20 | | 5.28 | | 4.74 | | 4.39 | | 4.10 | | 5.29 | | 4.76 | | 4.40 | | 4.11 | | 6.08 | | 5.47 | | 5.05 | | 4.72 | |  |
|  |  |  | | 25 | | 4.69 | | 4.22 | | 3.90 | | 3.65 | | 4.71 | | 4.23 | | 3.91 | | 3.65 | | 5.41 | | 4.86 | | 4.49 | | 4.20 | |  |
|  |  |  | | 30 | | 4.27 | | 3.84 | | 3.55 | | 3.32 | | 4.28 | | 3.85 | | 3.55 | | 3.32 | | 4.92 | | 4.42 | | 4.08 | | 3.82 | |  |
|  |  |  | | 35 | | 3.94 | | 3.54 | | 3.27 | | 3.06 | | 3.95 | | 3.55 | | 3.28 | | 3.07 | | 4.54 | | 4.08 | | 3.77 | | 3.52 | |  |
|  |  |  | | 40 | | 3.68 | | 3.31 | | 3.06 | | 2.86 | | 3.69 | | 3.32 | | 3.06 | | 2.86 | | 4.24 | | 3.81 | | 3.52 | | 3.29 | |  |
|  |  |  | | 50 | | 3.28 | | 2.95 | | 2.73 | | 2.55 | | 3.29 | | 2.96 | | 2.73 | | 2.56 | | 3.78 | | 3.4 | | 3.14 | | 2.93 | |  |
| Days | Old Sham SD | | Old MCAo SD | | N | | **Power %** | | | | | | | | | | | | | | | | | | | | | | | |
|  |  |  |  |  |  |  | **7 Days** | | | | | | | | **14 Days** | | | | | | | | **28 Days** | | | | | | | |
|  |  |  |  |  |  |  | **9.82** | | | | | | | | **12.49** | | | | | | | | **14.20** | | | | | | | |
| 7 | 3.24 | | 12.52 | |  |  | **95%** | | **90%** | | **85%** | | **80%** | | **95%** | | **90%** | | **85%** | | **80%** | | **95%** | | **90%** | | **85%** | | **80%** | |
| 14 | 3.13 | | 11.33 | | 3 | | 46.91 | | 42.18 | | 38.97 | | 36.44 | | 42.45 | | 38.17 | | 35.27 | | 32.97 | | 64.86 | | 58.32 | | 53.91 | | 50.41 | |
| 28 | 3.80 | | 17.32 | | 4 | | 37.73 | | 33.93 | | 31.34 | | 29.30 | | 34.14 | | 30.7 | | 28.36 | | 26.52 | | 52.16 | | 46.91 | | 43.36 | | 40.54 | |
|  |  | |  | | 5 | | 32.45 | | 29.18 | | 26.96 | | 25.20 | | 29.37 | | 26.41 | | 24.39 | | 22.81 | | 44.87 | | 40.34 | | 37.29 | | 34.87 | |
|  |  | |  | | 6 | | 28.91 | | 26 | | 24.02 | | 22.46 | | 26.16 | | 23.53 | | 21.74 | | 20.32 | | 39.97 | | 35.95 | | 33.23 | | 31.07 | |
|  |  | |  | | 7 | | 26.33 | | 23.68 | | 21.87 | | 20.45 | | 23.83 | | 21.43 | | 19.79 | | 18.51 | | 36.4 | | 32.73 | | 30.26 | | 28.29 | |
|  |  | |  | | 8 | | 24.34 | | 21.88 | | 20.22 | | 18.90 | | 22.02 | | 19.8 | | 18.29 | | 17.11 | | 33.65 | | 30.26 | | 27.97 | | 26.15 | |
|  |  | |  | | 9 | | 22.74 | | 20.45 | | 18.89 | | 17.66 | | 20.58 | | 18.5 | | 17.09 | | 15.98 | | 31.44 | | 28.27 | | 26.13 | | 24.43 | |
|  |  | |  | | 10 | | 21.42 | | 19.26 | | 17.79 | | 16.64 | | 19.38 | | 17.43 | | 16.10 | | 15.05 | | 29.61 | | 26.63 | | 24.61 | | 23.01 | |
|  |  | |  | | 12 | | 19.35 | | 17.4 | | 16.07 | | 15.03 | | 17.51 | | 15.75 | | 14.55 | | 13.60 | | 26.75 | | 24.06 | | 22.24 | | 20.79 | |
|  |  | |  | | 14 | | 17.78 | | 15.99 | | 14.77 | | 13.81 | | 16.09 | | 14.47 | | 13.37 | | 12.50 | | 24.59 | | 22.11 | | 20.44 | | 19.11 | |
|  |  | |  | | 16 | | 16.55 | | 14.88 | | 13.74 | | 12.85 | | 14.97 | | 13.46 | | 12.44 | | 11.63 | | 22.88 | | 20.57 | | 19.01 | | 17.78 | |
|  |  | |  | | 18 | | 15.54 | | 13.97 | | 12.91 | | 12.07 | | 14.06 | | 12.64 | | 11.68 | | 10.92 | | 21.48 | | 19.31 | | 17.85 | | 16.69 | |
|  |  | |  | | 20 | | 14.69 | | 13.21 | | 12.20 | | 11.41 | | 13.29 | | 11.95 | | 11.04 | | 10.33 | | 20.31 | | 18.26 | | 16.88 | | 15.78 | |
|  |  | |  | | 25 | | 13.06 | | 11.75 | | 10.85 | | 10.15 | | 11.82 | | 10.63 | | 9.82 | | 9.18 | | 18.06 | | 16.24 | | 15.01 | | 14.04 | |
|  |  | |  | | 30 | | 11.88 | | 10.68 | | 9.87 | | 9.23 | | 10.75 | | 9.67 | | 8.93 | | 8.35 | | 16.42 | | 14.77 | | 13.65 | | 12.76 | |
|  |  | |  | | 35 | | 10.97 | | 9.86 | | 9.11 | | 8.52 | | 9.93 | | 8.93 | | 8.25 | | 7.71 | | 15.16 | | 13.64 | | 12.60 | | 11.79 | |
|  |  | |  | | 40 | | 10.24 | | 9.21 | | 8.51 | | 7.95 | | 9.27 | | 8.33 | | 7.70 | | 7.20 | | 14.16 | | 12.73 | | 11.77 | | 11.00 | |
|  |  | |  | | 50 | | 9.13 | | 8.21 | | 7.59 | | 7.09 | | 8.26 | | 7.43 | | 6.87 | | 6.42 | | 12.63 | | 11.35 | | 10.49 | | 9.81 | |

| **Young animals** | | | | | |
| --- | --- | --- | --- | --- | --- |
| **Analysis:** | **A priori: Compute required sample size** | | **7 Days** | **14 Days** | **28 Days** |
| Input: | Tail(s) | = | Two | Two | Two |
|  | Effect size d | = | 74.244.466 | 53.589.228 | 36.181.918 |
|  | α err prob | = | 0.05 | 0.05 | 0.05 |
|  | Power (1-β err prob) | = | 0.95 | 0.95 | 0.95 |
|  | Allocation ratio N2/N1 | = | 1 | 1 | 1 |
| Output: | Noncentrality parameter δ | = | 90.930.529 | 65.633.132 | 51.168.959 |
|  | Critical t | = | 27.764.451 | 27.764.451 | 24.469.119 |
|  | Df | = | 4 | 4 | 6 |
|  | Sample size group 1 | = | 3 | 3 | 4 |
|  | Sample size group 2 | = | 3 | 3 | 4 |
|  | Total sample size | = | 6 | 6 | 8 |
| Actual power | = |  | 0.99 | 0.99 | 0.98 |

| **Old animals** | | | | | |
| --- | --- | --- | --- | --- | --- |
| **Analysis:** | **A priori: Compute required sample size** | | **7 Days** | **14 Days** | **28 Days** |
| Input: | Tail(s) | = | Two | Two | Two |
|  | Effect size d | = | 10.738.559 | 10.738.559 | 10.738.559 |
|  | α err prob | = | 0.05 | 0.05 | 0.05 |
|  | Power (1-β err prob) | = | 0.95 | 0.95 | 0.95 |
|  | Allocation ratio N2/N1 | = | 1 | 1 | 1 |
| Output: | Noncentrality parameter δ | = | 37.199.460 | 37.199.460 | 37.199.460 |
|  | Critical t | = | 20.128.956 | 20.128.956 | 20.128.956 |
|  | Df | = | 46 | 46 | 46 |
|  | Sample size group 1 | = | 24 | 24 | 24 |
|  | Sample size group 2 | = | 24 | 24 | 24 |
|  | Total sample size | = | 48 | 48 | 48 |
| Actual power | = |  | 0.95 | 0.95 | 0.95 |
